# 2′-Fluoro-c-di-GMP as an oral vaccine adjuvant†

**DOI:** 10.1039/c9ra08310c

**Published:** 2019-12-16

**Authors:** Jia Li, Rhonda Kuo Lee, Wangxue Chen, Hongbin Yan

**Affiliations:** Department of Chemistry, Brock University 1812 Sir Isaac Brock Way St. Catharines ON L2S 3A1 Canada tyan@brocku.ca; Human Health and Therapeutics Research Center, National Research Council of Canada 100 Sussex Dr Ottawa ON K1A 0R6 Canada; Department of Biological Sciences, Brock University 1812 Sir Isaac Brock Way St. Catharines ON L2S 3A1 Canada

## Abstract

Bis-(3′–5′)-cyclic dimeric 2′-deoxy-2′-fluoroguanosine monophosphate (2′-F-c-di-GMP) was synthesized through the modified H-phosphonate chemistry. Oral immunization of C57BL/6 mice with *Helicobacter pylori* cell-free sonicate extract adjuvanted with 2′-F-c-di-GMP led to the production of antigen-specific antibodies in feces and sera, and lowered bacterial counts in the stomach upon post-vaccination infections in immunized mice. Similarly, oral vaccination of BALB/c mice with flagillin proteins from *Clostridium difficile* and *Listeria monocytogenes* adjuvanted with 2′-F-c-di-GMP led to production of antigen-specific antibodies both systemically and mucosally. The adjuvanticity of 2′-F-c-di-GMP is associated with the enhanced induction of interferon γ. These results demonstrated the excellent oral adjuvanticity of 2′-F-c-di-GMP.

## Abstract 2

2′-F-c-di-GMP was synthesized through the modified H-phosphonate chemistry. 2′-F-c-di-GMP was found to be an effective mucosal vaccine adjuvant, both intranasally and orally.

## Graphical abstract

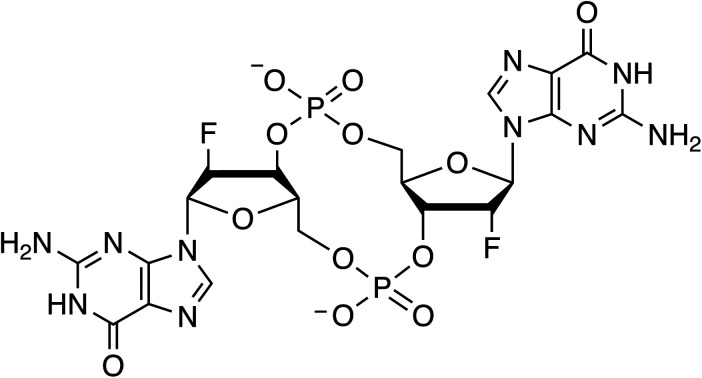

## Introduction

In the last decade or so, 3′,5′-cyclic diguanylic acid (c-di-GMP) has been recognized as a potent immunostimulator and a useful mucosal adjuvant in a number of models.^1,2^ It was previously demonstrated by us that intranasal administration of c-di-GMP prior to bacterial challenges provides mice with protection against *Acinetobacter baumannii* infection by chemokine induction and enhanced neutrophil recruitment.^3^ Furthermore, we showed that intranasal immunization of mice with pneumococcal surface adhesion A (PsaA) adjuvanted with c-di-GMP invoked strong antigen-specific serum immunoglobulin G (IgG) and secretory IgA antibody responses, and the nasopharyngeal *Streptococcus pneumoniae* colonization in immunized mice was significantly reduced.^4^ In the present study, we wish to demonstrate the adjuvanticity of c-di-GMP and its 2′-fluoro-analog (2′-F-c-di-GMP) in oral immunization of mice against *Helicobacter pylori*. In this respect, fluorine atoms are small and electronegative. Incorporation of fluorine at the 2′-position of nucleosides is an effective approach to modulate sugar puckers.^5^ Furthermore, introduction of fluorine into therapeutic agents has been well recognized as a useful modification to modulate pharmacological properties.^6–9^

We report herein that oral immunization of C57BL/6 mice with *H. pylori* cell-free sonicate extract (HPCE) adjuvanted with 2′-F-c-di-GMP led to the production of antigen-specific antibodies, and provide excellent protective immunity of immunized mice against *H. pylori* challenges. In a similar manner, productions of antigen-specific antibodies were also demonstrated in mice immunized with flagillin proteins from Gram-positive bacterium *Clostridium difficille* and intracellular pathogen *Listeria monocytogenes*.

## Results and discussion

### Synthesis of 2′-F-c-di-GMP *via* the modified H-phosphonate chemistry

**Scheme 1:**
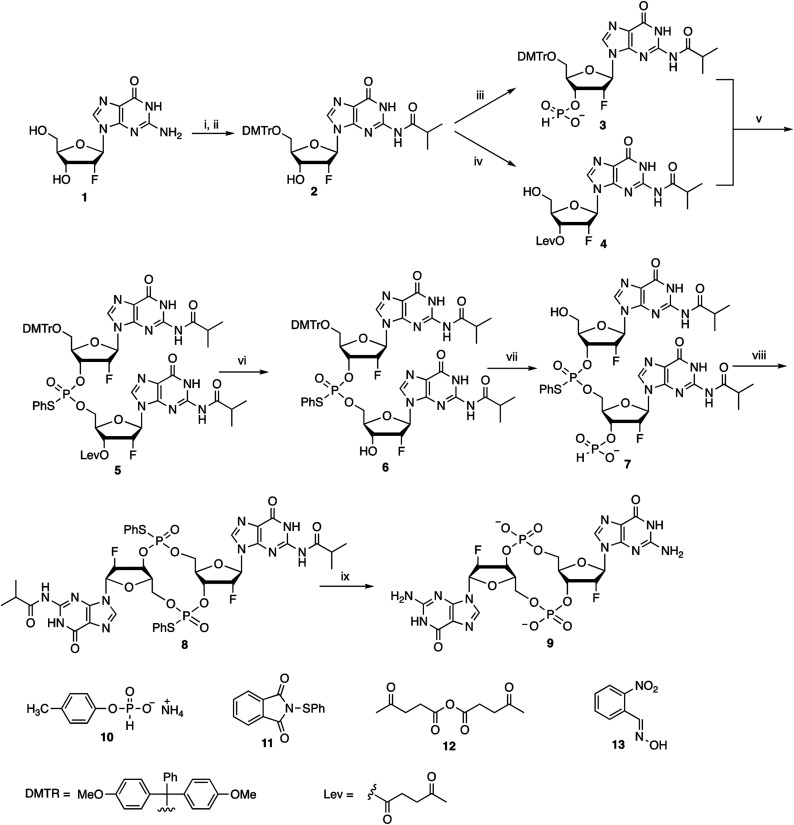
Reagents and conditions: (i) (a) (CH_3_)_3_SiCl, (CH_3_)_2_CHCOCl, C_5_H_5_N; (b) aq. NH_3_; (ii) DMTrCl, C_5_H_5_N; (iii) (a) 10, (CH_3_)_3_CCOCl, C_5_H_5_N; (b) H_2_O; (iv) (a) 12, C_5_H_5_N; (b) Cl_2_CHCOOH, pyrrole, CH_2_Cl_2_; (v) (a) (CH_3_)_3_CCOCl, C_5_H_5_N; (b) 11, C_5_H_5_N; (vi) NH_2_NH_2_·H_2_O, CH_3_COOH, C_5_H_5_N, H_2_O; (vii) (a) 10, (CH_3_)_3_CCOCl, C_5_H_5_N; (b) H_2_O; (c) Cl_2_CHCOOH, pyrrole, CH_2_Cl_2_; (viii) (a) (PhO)_2_P(O)Cl, C_5_H_5_N; (b) 11, C_5_H_5_N; (ix) (a) 13, TMG; (b) aq. NH_3_, 55 °C.

We previously demonstrated the synthesis of c-di-GMP *via* the modified H-phosphonate chemistry.^10,11^ In a similar manner, the synthesis of 2′-F-c-di-GMP started with protecting the exocyclic amino residues of 2′-deoxy-2′-fluoro-guanosine 1 with isobutyryl group. The resulting *N*-isobutyryl-2′-deoxy-2′-fluoro-guanosine was then protected with dimethoxytrityl (DMTr) group at the 5′-OH position. Thus, 5′-*O*-DMTr-2-*N*-isobutyryl-2′-deoxy-2′-fluoro-guanosine 2 was obtained in 79% overall yield in two steps (steps (i) and (ii), Scheme 1). The building blocks H-phosphonate triethylammonium salt 3 and 3′-*O*-levulinyl (Lev) nucleoside 4 were prepared according to the process described in Scheme 1.

In the modified H-phosphonate approach the fully protected linear dimer phosphorothioate triester 5 was prepared by reacting H-phosphonate triethylammonium salt 3 and 3′-*O*-Lev nucleoside 4 in the presence of pivaloyl chloride followed by oxidation with 1-phenylsulfanyl-pyrrolidine-2,5-dione 11. After removal of 3′-*O*-levulinyl group from the dimer 5 by the treatment with hydrazine hydrate in pyridine–acetic acid solution, the product was transformed into the corresponding linear dimer H-phosphonate triethylammonium salt, followed by removal of 5′-*O*-dimethoxytrityl group. The resulting linear dimer H-phosphonate 7 was then subjected to cyclization under high dilution conditions to give the fully protected 2′-deoxy-2′-fluoro cyclic diguanylic acid 8 in 59% yield.

Two steps were involved in the deprotection of the fully protected 2′-fluoro cyclic diguanylic acid 8 (step (ix), Scheme 1), treatment with 2-nitrobenzaldoxime 13 in the present of *N*,*N*,*N*′,*N*′-tetramethylguanidine (TMG) followed by amminolysis. The fully unprotected bis-(3′–5′)-cyclic dimeric 2′-deoxy-2′-fluoroguanosine monophosphate 9 was characterized by ^1^H and ^31^P NMR (Fig. S1†). The reverse phase HPLC profile confirmed the homogeneity of the product (Fig. S2†). The molecular mass of the product was found by ESI to be 693.1 as [M − H]^−^ which is in agreement with the calculated value (693.1) (Fig. S3†).

### Intranasally administered 2′-F-c-di-GMP induces strong antigen-specific antibody responses in the serum and at multiple mucosal sites

**Fig. 1:**
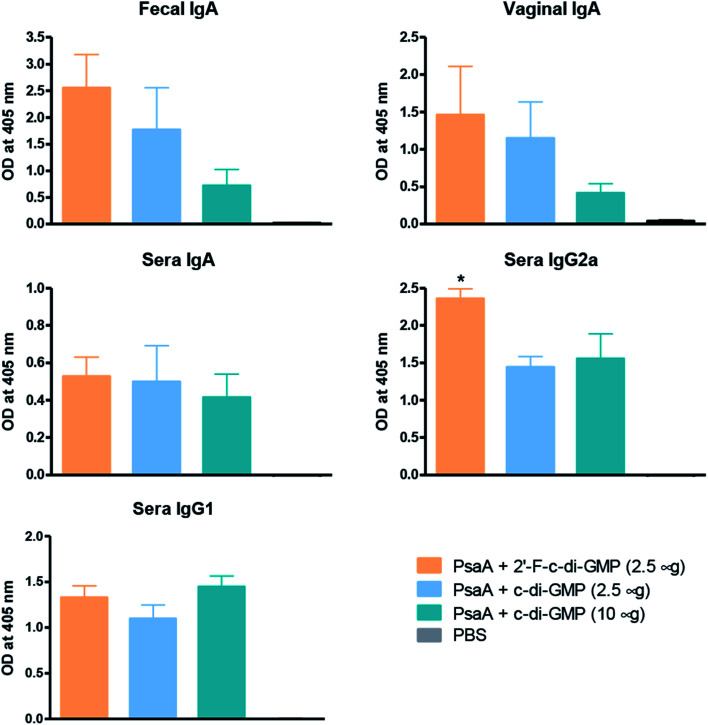
Induction of antigen-specific mucosal IgA responses by intranasal administration of 2′-F-c-di-GMP. Groups of 5 BALB/c mice were intranasally immunized with 2 μg PsaA admixed with 2.5 μg 2′-F-c-di-GMP, 2.5 μg c-di-GMP or 10 μg c-di-GMP at day 0, 14 and 21. Additional group of mice were immunized with phosphate-buffered saline (PBS) and served as sham-immunized group. The feces, vaginal washing and blood samples were collected at day 28 and assayed by ELISA for PsaA-specific IgA and IgG isotypes (IgG1 and IgG2a) responses. *P < 0.05 *vs.* PsaA + c-di-GMP groups.

We and others have previously shown that c-di-GMP is a potent mucosal adjuvant when administered intranasally.^4,12–14^ In this study, we first determined if i.n. immunization of mice with 2′-F-c-di-GMP can elicit antigen specific mucosal immune responses at a comparable magnitude to those induced by the parental c-di-GMP. As shown in Fig. 1, co-administration of pneumococcal protein PsaA with 2′-F-c-di-GMP induced higher levels of PsaA-specific IgA in feces and vaginal wash, and serum IgG2a than the co-administration with c-di-GMP whereas the serum PsaA-specific IgA and IgG1 levels were comparable between the two adjuvants. As expected, sham-immunized mice showed no specific antibody responses in the serum or mucosal samples.

**Fig. 2:**
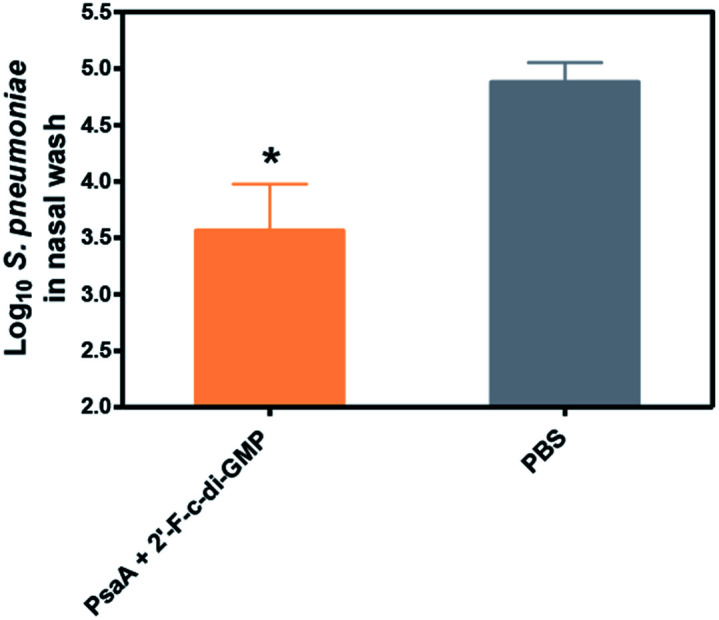
Reduced nasopharyngeal colonization by *S. pneumoniae* in mice intranasally immunized with 2′-F-c-di-GMP-adjuvanted vaccine. Groups of 5 BALB/c mice were intranasally immunized with 2 μg PsaA admixed with 2.5 μg 2′-F-c-di-GMP or sham-immunized with PBS at day 0, 14 and 21. The mice were intranasally challenged at day 35 with 5 × 10^6^ CFU type 14 *S. pneumoniae* and the bacterial numbers in the nasal cavity of challenged mice were determined 3 days later. *P < 0.05 *vs.* PBS group.

More importantly, we found that the mucosal immune responses induced by the i.n. immunization with 2′-F-c-di-GMP adjuvanted vaccine were protective against mucosal infections in the well-established mouse *S. pneumoniae* colonization model (Fig. 2) in that mice i.n. immunized with PsaA + 2′-F-c-di-GMP showed significantly reduced colonization of *S. pneumoniae* when compared to sham-immunized mice (*P* < 0.05). The magnitude of this reduction was comparable to that attained in mice immunized with PsaA adjuvanted with cholera toxin (CT),^4^ the golden standard of mucosal adjuvant which has undesirable toxicity for human applications. We have previously shown that immunization with PsaA alone at this dose showed no effect on the bacterial colonization.^4^ These results demonstrated that 2′-F-c-di-GMP is a potent mucosal adjuvant when administered by intranasal route, and that 2′-F-c-di-GMP induces a potent, protective immunity against i.n. challenge with *S. pneumoniae* when co-administered with the PsaA antigen *via* i.n. route. Therefore, further exploration of this molecule as a potential mucosal adjuvant is warranted.

### Oral immunization with 2′-F-c-di-GMP-adjuvanted vaccine induces strong antigen-specific antibody responses in the serum and at multiple mucosal sites

**Fig. 3:**
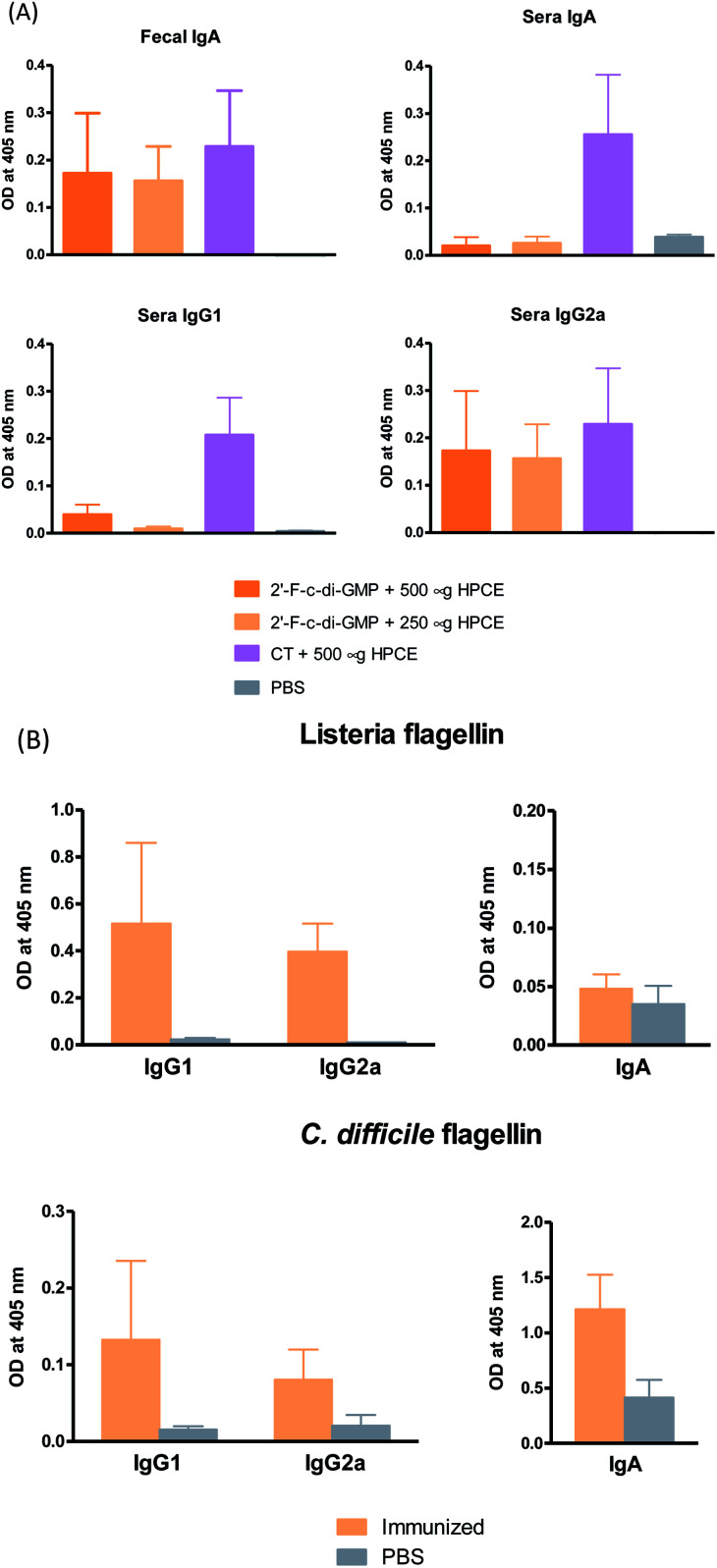
Induction of antigen-specific mucosal IgA responses by oral administration of 2′-F-c-di-GMP. Groups of 5 C56BL/6 mice were orally immunized with varying amount of *H. pylori* cell free sonicate extract (HPCE) (A) or flagillin proteins from *C. difficile* and *L. monocytogenes* (B) admixed with either 100 μg 2′-F-c-di-GMP or 10 μg cholera toxin (CT, positive control) at day 0, 14 and 21. Additional group of mice were immunized with PBS and served as sham-immunized group. Feces and blood samples were collected at day 28 and assayed by ELISA for antigen-specific IgA and IgG isotypes (IgG1 and IgG2a) responses.

Despite the well-recognized socioeconomic and safety advantages of oral immunization over the parenteral or i.n. immunization, only a limited number of oral vaccines are currently approved for human use.^15^ Oral vaccination is the most challenging vaccination method due to the administration route. Indeed, we found that oral administration of the parental c-di-GMP as a mucosal adjuvant failed to induce reliable mucosal or systemic immune responses (unpublished data). In this study, we therefore assessed if oral administration of 2′-F-c-di-GMP induces antigen-specific mucosal immune responses. As shown in Fig. 3, oral co-administration of both high and low doses of HPCE with 2′-F-c-di-GMP induced substantial amount of antigen-specific fecal IgA and serum IgG2a responses, which were similar in the magnitude to those induced by CT (Fig. 3A). As expected, sham-immunized mice showed no specific antibody responses in the serum or fecal samples. Similarly, oral co-administration of 2′-F-c-di-GMP with flagellin antigens purified from *L. monocytogenes* (50 μg) or *C. difficile* (30 μg) induced substantial amount of *C. difficile* flagellin-specific IgA and small amount of *Listeria* flagellin-specific IgA in feces as well as serum IgG1 and IgG2a responses, as compared with sham-immunized mice (Fig. 3B). These results demonstrated that 2′-F-c-di-GMP enhances mucosal immune responses to microbial antigens when administered *via* the oral route, and indicate that 2′-F-c-di-GMP can be used in oral vaccines as a potent mucosal adjuvant.

### Oral immunization of mice with 2′-F-c-di-GMP-adjuvanted HPCE significantly reduces gastric colonization by *H. pylori*

**Fig. 4:**
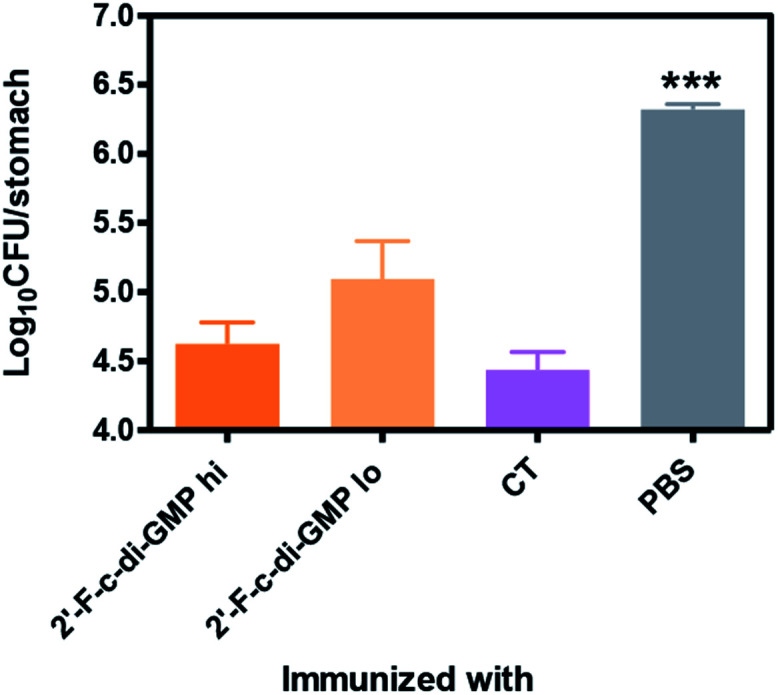
Reduced gastric colonization by *H. pylori* in mice orally immunized with 2′-F-c-di-GMP-adjuvanted vaccine. Groups of 5 C56BL/6 mice were orally immunized with varying amount of *H. pylori* cell free sonicate extract (HPCE) admixed with either 100 μg 2′-F-c-di-GMP or 10 μg cholera toxin (CT, positive control) at day 0, 14 and 21. Additional group of mice were immunized with PBS and served as sham-immunized group. The mice were orally challenged 3× between day 35 and 42 with 10^8^ CFU *H. pylori* SS1 and the bacterial numbers in the gastric mucosa of challenged mice were determine 4 weeks later. ***P < 0.001 *vs.* immunized groups.

We next determined if the mucosal immune responses induced by the 2′-F-c-di-GMP adjuvanted *H. pylori* oral vaccine protect against *H. pylori* challenge in a mouse model of *H. pylori* infection.^16^ As shown in Fig. 4, quantitative bacteriology showed that oral immunization of mice with both low (250 μg) and high (500 μg) doses of *H. pylori* HPCE + 2′-F-c-di-GMP vaccines significantly reduced the bacterial burdens in the gastric mucosa at 4 weeks post-challenge when compared to sham-immunized mice (*P* < 0.001). Moreover, the magnitude of this reduction was comparable to that attained in mice immunized with HPCE adjuvanted with CT. As anticipated, immunization of mice with HPCE alone failed to reduce the bacterial colonization. These results suggest that 2′-F-c-di-GMP is capable of inducing protective mucosal immunity against mucosal pathogens upon oral immunization with specific antigen.

### Oral immunization with 2′-F-c-di-GMP-adjuvanted vaccine induces antigen-specific Th1/Th17 cytokine responses

**Fig. 5:**
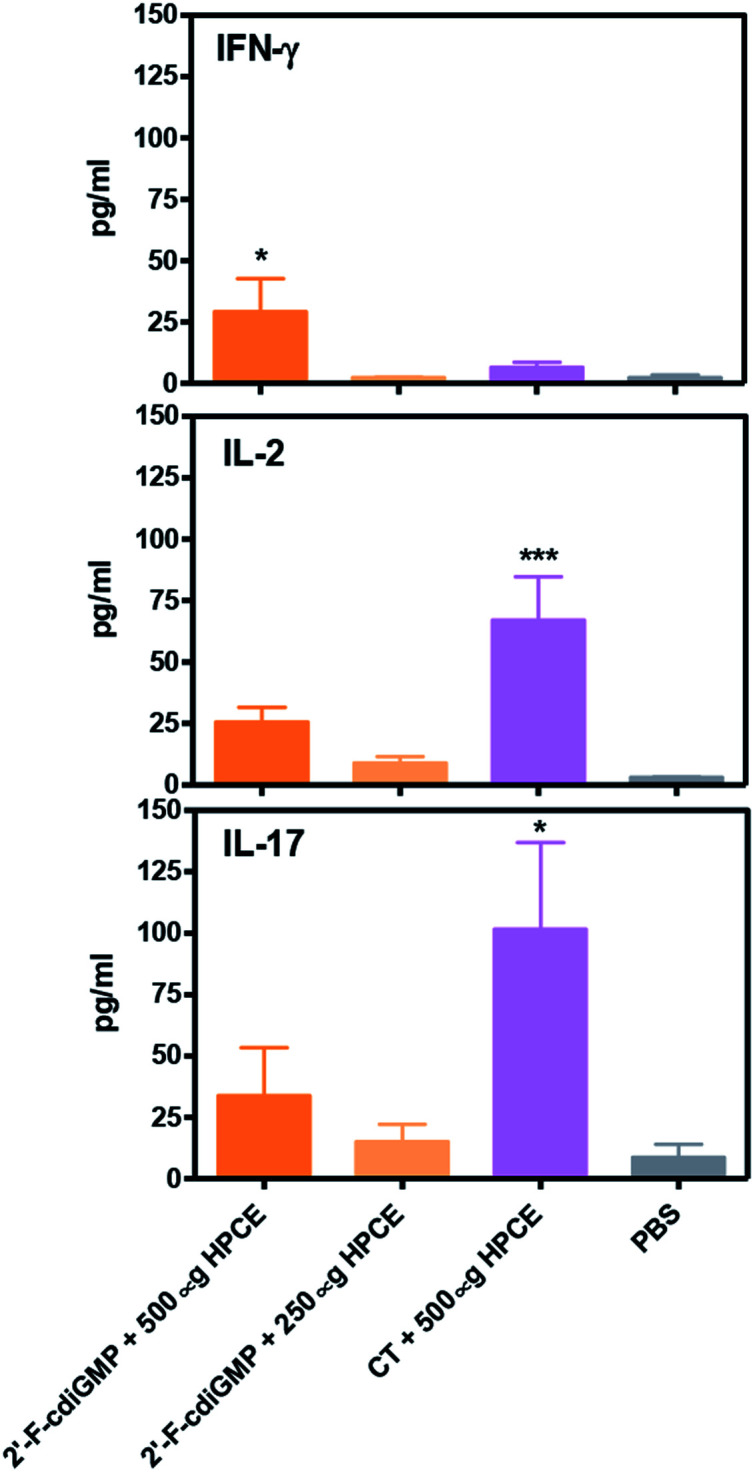
Induction of antigen-specific Th1/Th17 cytokine responses by oral administration of 2′-F-c-di-GMP. Groups of 5 C56BL/6 mice were orally immunized with varying amount of *H. pylori* cell free sonicate extract (HPCE) admixed with either 100 μg 2′-F-c-di-GMP or 10 μg cholera toxin (CT, positive control) at day 0, 14 and 21. Additional group of mice were immunized with PBS and served as sham-immunized group. The spleens were collected at day 28 and single cell suspension was prepared for the determination of antigen-specific cytokine responses. The cells were stimulated by 10 mg ml^−1^ HPCE and cultured at 37 °C in a 5% CO_2_ atmosphere for 72 hours. At the end of the culture, the supernatant was collected and assayed for the levels of IFN-γ, IL-2 and IL-17 by Luminex. *P < 0.05 and ***P < 0.001 *vs.* PBS group.

Previous studies by others have implied that Th1/Th17 immune responses may play an important role in host defence against *H. pylori* infection.^17^ We next examined if oral administration of 2′-F-c-di-GMP + HPCE induced antigen-specific Th1/Th17 cytokine responses. Compared to sham-immunized mice, the splenocytes from 2′-F-c-di-GMP immunized mice produced substantially higher amount of IFN-γ, IL-2 and IL-17 in response to HPCE stimulation (Fig. 5). The amount of IFN-γ produced by the splenocytes from 2′-F-c-di-GMP immunized mice was even higher than that produced by the cells from CT-immunized mice although the latter mice produced higher amount of IL-2 and IL-17 than the former mice. These results indicate that the protection against *H. pylori* infection induced by oral immunization with 2′-F-c-di-GMP in mice is associated with the production of antigen-specific IFN-γ.

## Experimental

### Synthesis of compounds

#### 2′-Fluoro-5′-*O*-dimethoxytrityl-2-*N*-isobutyl-2′-deoxyguanosine 2

2′-Fluoro-2′-deoxyguanosine 1 (1.00 g, 3.51 mmol) was dried *in vacuo* at 60 °C for 5 h followed by addition of dry pyridine (10.0 ml). After the solution was cooled (ice-water bath), chlorotrimethylsilane (2.23 ml, 17.6 mmol) was added and the reaction mixture was stirred for 30 min. The mixture was then evaporated to *ca*. half of the original volume. To the residue, dry pyridine (5.0 ml) was added and mixture was cooled (ice-water bath) followed by dropwise addition of isobutyric anhydride (3.02 ml, 18.2 mmol). After 3 h, the reaction was quenched by addition of water (3.0 ml) followed after 15 min aqueous ammonium hydroxide (30–33%, 10.0 ml). The products were first evaporated under reduced pressure while the temperature was kept below 10 °C. After the bulk ammonia was removed, the products were evaporated to dryness under reduced pressure while the temperature was kept below 35 °C. The residue was co-evaporated with dry pyridine (2 × 10 ml) and then dissolved in dry pyridine (15.0 ml) followed by addition of 4,4′-dimethoxytrityl chloride (1.15 g, 3.39 mmol). Water (2.0 ml) was added after 30 min to quench the reaction. The products were concentrated under reduced pressure and the residue was dissolved in dichloromethane (25 ml) and extracted with saturated aqueous sodium hydrogen carbonate (15 ml). The layers were separated, and the aqueous layer was back extracted with dichloromethane (2 × 5 ml). The combined organic layers were dried (MgSO_4_) and concentrated with a rotary evaporator. The residue was purified by column chromatography on silica gel. The appropriate fractions, which were eluted with dichloromethane–methanol (98 : 2 v/v) containing 0.5% triethylamine, were concentrated under reduced pressure to give the title compound as a colourless glass (1.83 g, 79%). *δ*_H_[(CD_3_)_2_SO]: 12.15 (1H, s, NH, ex), 11.65 (1H, s, NH, ex), 8.13 (1H, s, H-8), 7.34–7.19 (9H, m, Ar–H), 6.83–6.78 (4H, m, Ar–H), 6.21 (1H, d, *J* = 18.7, H-1′), 5.67 (1H, d, *J* = 7.2, 3′-OH, ex), 5.42 (1H, dd, *J* = 52.9 and 4.6, H-2′), 4.67–4.53 (1H, m, H-3′), 4.12 (1H, m, H-4′), 3.71 (3H, s, –OCH_3_), 3.72 (3H, s, –OCH_3_), 2.76 (1H, CH, m), 1.12 (3H, d, *J* = 7.0, CH_3_), 1.11 (3H, d, *J* = 7.0, CH_3_). *R*_f_: 0.46 (dichloromethane–methanol 95 : 5, v/v).

#### 2′-Fluoro-3′-*O*-levulinyl-2-*N*-isobutyryl-2′-deoxyguanosine 4

2′-Fluoro-5′-*O*-dimethoxytrityl-2-*N*-isobutyryl-2′-deoxyguanosine 2 (1.00 g, 1.52 mmol) was co-evaporated with dry toluene (2 × 5 ml) and then dissolved in dry pyridine (10.0 ml) followed by addition of levulinic anhydride (0.65 g, 3.04 mmol). After 16 h, water (3.0 ml) was added and the solution was concentrated under reduced pressure. The residue was dissolved in dichloromethane (25 ml) and extracted with saturated aqueous sodium hydrogen carbonate (15 ml). The layers were separated and the aqueous layer was back extracted with dichloromethane (2 × 5 ml). The combined organic layers were dried (MgSO_4_) and evaporated under reduced pressure. The residue was co-evaporated with dry toluene (3 × 5 ml) and dissolved in dry dichloromethane (10.0 ml). Distilled pyrrole (1.05 ml, 15.1 mmol) followed by dichloroacetic acid (0.56 ml, 6.79 mmol) were added. After 10 min, the products were extracted with saturated aqueous sodium hydrogen carbonate (15 ml). The layers were separated and the aqueous layer was back extracted with dichloromethane (2 × 5 ml). The combined organic layers were dried (MgSO_4_) and concentrated under reduced pressure. The residue was purified by column chromatography on silica gel. The appropriate fractions, which were eluted with dichloromethane–methanol (97 : 3 v/v), were combined and concentrated under reduced pressure to give the title compound as a colourless glass (0.51 g, 74%). ESI-MS found [M − H]^−^ = 452.1, C_19_H_23_FN_5_O_7_^−^ requires 452.158. *δ*_H_[(CD_3_)_2_SO]: 12.14 (1H, s, NH, ex), 11.69 (1H, s, NH, ex), 8.31 (1H, s, H-8), 6.17 (1H, dd, *J* = 14.9 and 4.2 Hz, H-1′), 5.69 (1H, ddd, *J* = 51.0, 4.5 and 4.5, H-2′), 5.43–5.35 (2H, m, H-3′ and 5-OH), 4.23 (1H, d, *J* = 3.9 Hz, H-4′), 3.76–3.59 (2H, m, H-5′, H-5′′), 2.82–2.75 (3H, m, CH_2_ and CH), 2.62–2.57 (2H, m, CH_2_), 2.13 (3H, s, CH_3_), 1.12 (6H, d, *J* = 6.8, 2×CH_3_). *δ*_F_[(CD_3_)_2_SO]: −206.2 (ddd, *J* = 51.1, 11.7 and 12.3). *R*_f_: 0.31 (dichloromethane–methanol 95 : 5, v/v).

#### 2′-Fluoro-5′-*O*-dimethoxytrityl-2-*N*-isobutyryl-2′-deoxyguanosine 3′-H-phosphonate triethylammonium salt 3

Ammonium *p*-tolyl H-phosphonate (0.95 g, 5.03 mmol) was co-evaporated with triethylamine (1.40 ml, 10.0 mmol) and methanol (8.0 ml) under reduced pressure. To the residue was added 2′-fluoro-5′-*O*-dimethoxytrityl-2-*N*-isobutyryl-2′-deoxyguanosine 2 (1.10 g, 1.67 mmol), and the mixture was co-evaporated with dry pyridine (2 × 5 ml) and then dissolved in dry pyridine (25 ml). To the cooled (ice-water bath) mixture was added dropwise pivaloyl chloride (0.68 ml, 5.55 mmol) over 5 min. After 1 h water (5.0 ml) was added. After a further period of 1 h at room temperature, the products were concentrated under reduced pressure and the residue was dissolved in dichloromethane (25 ml) and extracted with saturated aqueous sodium hydrogen carbonate (15 ml). The layers were separated and the aqueous layer was back extracted with dichloromethane (2 × 5 ml). The combined organic layers were extracted with triethylammonium phosphate buffer (20 ml, 0.5 *M*, pH 7.0) and the organic layer was back extracted by dichloromethane (15 ml). The combined organic layers were dried (MgSO_4_) and concentrated under reduced pressure. The residue was purified by short column chromatography on silica gel. The appropriate fractions, which were eluted with dichloromethane–methanol (90 : 10 v/v), were pooled and concentrated under reduced pressure to give triethylammonium salt of the title compound as a colourless glass (1.18 g, 86%). ESI-MS found M^−^ = 720.2, C_35_H_36_FN_5_O_9_P^−^ requires 720.2. *δ*_H_[(CD_3_)_2_SO]: 12.37 (1H, s, NH, ex), 11.84 (1H, s, NH, ex), 8.13 (1H, s, H-8), 7.21–7.10 (9H, m, Ar), 6.78 (4H, dd, *J* = 3.3 and 9.0, Ar), 6.21 (1H, d, *J* = 18.4, H-1′), 5.79 (1H, d, *J* = 54.0, H-2′), 4.12 (1H, br, H-3′), 3.70 (6H, s), 3.07 (1H, m, H-4′), 2.92 (6H, q, *J* = 7.0), 2.73 (1H, sep, *J* = 7.0, CH), 1.10 (15H, m). *δ*_P_[(CD_3_)_2_SO]: −0.72 (dd, *J*_PH_ = 589.5, *J*_PC_ = 10.4). *δ*_F_[(CD_3_)_2_SO]: −203.15 (ddd, *J* = 18.8, 24.7 and 54.2).

#### DMTr-*G*′*p*(*s*′)*G*′-Lev 5

2′-Fluoro-3′-*O*-levulinyl-2-*N*-isobutyryl-2′-deoxyguanosine 4 (0.44 g, 0.97 mmol) and 2′-fluoro-5′-*O*-dimethoxytrityl-2-*N*-isobutyryl-2′-deoxyguanosine 3′-H-phosphonate triethylammonium salt 3 (1.00 g, 1.22 mmol) were co-evaporated with dry pyridine (2 × 5 ml) and then dissolved in dry pyridine (5.0 ml) and cooled (ice-water bath). Pivaloyl chloride (0.31 ml, 2.53 mmol) followed by 1-phenylsulfanyl-pyrrolidine-2,5-dione 11 (0.56 g, 2.20 mmol) were added after 5 min. The reaction mixture was stirred for 30 min at room temperature, and then water (0.2 ml) was added. After 10 min the products were concentrated under reduced pressure. The residue was dissolved in dichloromethane (25 ml) and extracted with saturated aqueous sodium hydrogen carbonate (15 ml). The layers were separated and the aqueous layer was back extracted with dichloromethane (2 × 5 ml). The combined organic layers were dried (MgSO_4_) and concentrated under reduced pressure. The residue was purified by column chromatography on silica gel. The appropriate fractions, which were eluted with dichloromethane–methanol (98 : 2 v/v) containing 0.5% of triethylamine, were pooled and concentrated under reduced pressure to give the title compound as a colourless glass (1.35 g, 109%. The yield is over 100%, due to inseparable impurities, likely phthalimide). ESI-MS found [M − H]^−^ = 1263.7, C_60_H_62_F_2_N_10_O_15_PS^−^ requires 1263.38. *δ*_P_[(CD_3_)_2_SO]: 23.79, 23.02. *R*_f_: 0.39 (dichloromethane–methanol 95 : 5, v/v).

#### DMTr-*G*′*p*(*s*′)*G*′-OH 6

DMTr-*G*′*p*(s′)*G*′-Lev 5 (1.20 g, 0.95 mmol) was dissolved in pyridine (10.0 ml) followed by addition of a mixture of hydrazine monohydrate (0.40 ml, 8.24 mmol), acetic acid (4.8 ml), water (0.8 ml) and pyridine (18.0 ml). The reaction mixture was stirred at room temperature for 15 min followed by addition of pentane-2,4-dione (1.20 ml). After 10 min the products were concentrated under reduced pressure. The residue was dissolved in dichloromethane (25 ml) and extracted with saturated aqueous sodium hydrogen carbonate (15 ml). The layers were separated and the aqueous layer was back extracted with dichloromethane (2 × 5 ml). The combined organic layers were dried (MgSO_4_) and concentrated under reduced pressure. The residue was purified by column chromatography on silica gel. The appropriate fractions, which were eluted with dichloromethane–methanol (95 : 5 v/v), were concentrated under reduced pressure to give the title compound as a colourless solid (1.05 g, 95%). ESI-MS found for [M − H]^−^ 1166.3, C_55_H_57_F_2_N_10_O_13_PS^−^ requires 1166.35. *δ*_P_[(CD_3_)_2_SO]: 23.68, 22.96. *R*_f_: 0.27 (dichloromethane–methanol 95 : 5, v/v).

#### HO-*G*′*p*(s′)*G*′p(H) 7

Ammonium *p*-tolyl H-phosphonate 10 (0.57 g, 3.01 mmol) was co-evaporated with triethylamine (0.84 ml, 6.03 mmol) and methanol (5.3 ml). To the residue was added DMTr-*G*′*p*(*s*′)*G*′-OH 6 (1.03 g, 0.88 mmol) and the mixture was co-evaporated with dry pyridine (2 × 5 ml) and then dissolved in more dry pyridine (10.0 ml). To this cooled (ice-water bath) reaction mixture was added pivaloyl chloride (0.37 ml, 3.0 mmol) over a period of 5 min. After 30 min water (1.0 ml) was added. The products were allowed to warm up to room temperature and stirring was continued for another 1 h. The products were concentrated under reduced pressure and the residue was dissolved in dichloromethane (25 ml) and extracted with saturated aqueous sodium hydrogen carbonate (15 ml). The layers were separated and the aqueous layer was back extracted with dichloromethane (2 × 5 ml). The combined organic layers were dried (MgSO_4_) and concentrated under reduced pressure. The residue was co-evaporated with dry toluene (3 × 10 ml) and then dissolved in dry dichloromethane (20 ml). Freshly distilled pyrrole (1.04 ml, 15.0 mmol) and dichloroacetic acid (0.86 ml, 10.4 mmol) were added. The reaction mixture was stirred for 10 min and then extracted with saturated aqueous sodium hydrogen carbonate (15 ml). The layers were separated and the aqueous layer was back extracted with dichloromethane (2 × 5 ml). The combined organic layers were extracted with triethylammonium phosphate buffer (20 ml, pH 7.0, 0.5 M) and the aqueous layer was back extracted by dichloromethane (15 ml). The combined organic layers were dried (MgSO_4_) and concentrated under reduced pressure. The residue was purified by short column chromatography on silica gel. The appropriate fractions, eluted with dichloromethane–methanol (90 : 10 v/v), were concentrated under reduced pressure to give triethylammonium salt of HO-*G*′*p*(*s*′)*G*′p(H) as a colourless glass (367 mg, 40%). ESI-MS found [M − H]^−^ = 927.3, C_34_H_39_F_2_N_10_O_13_P_2_S^−^ requires 927.187. *δ*_P_[(CD_3_)_2_SO]: 23.46 and 21.82 (1 P), 0.32 and 0.17 (1 P, dd, *J*_PH_ = 602.6 Hz, *J*_PC_ = 11.4 Hz).

#### Preparation of fully protected 2′-fluoro-c-di-GMP 8

HO-*G*′*p*(*s*′)*G*′p(H) triethylammonium salt 7 (100 mg, 0.097 mmol) was co-evaporated with dry pyridine (2 × 3 ml) and then dissolved in dry dichloromethane (5.0 ml). This solution was added to a solution of diphenyl chlorophosphate (0.41 ml, 1.98 mmol) in dry pyridine (5.0 ml) dropwise over 20 min while the temperature was kept at −40 °C (dry ice-acetone bath). After 20 min 1-phenylsulfanyl-pyrrolidine-2,5-dione 11 (0.45 g, 1.76 mmol) was added and the reaction mixture was allowed to warm up to room temperature. Stirring was continued for another 30 min and then water (0.5 ml) was added. After 5 min the products were concentrated under reduced pressure. The residue was dissolved in dichloromethane (25 ml) and extracted with saturated aqueous sodium hydrogen carbonate (15 ml). The layers were separated and the aqueous layer was back extracted with dichloromethane (2 × 5 ml). The combined organic layers were dried (MgSO_4_) and concentrated under reduced pressure. The residue was purified by short column chromatography on silica gel. The appropriate fractions, which were eluted with dichloromethane–methanol (97 : 3 v/v), were combined and concentrated under reduced pressure to give the fully protected 2′-fluoro-c-di-GMP 8 as a colourless glass (58 mg, 59%). ESI-MS found [M + H]^+^ = 1019.2, C_40_H_43_F_2_N_10_O_12_P_2_S_2_^+^ requires 1019.19. *δ*_P_[(CD_3_)_2_SO]: 24.50, 22.98, 21.60. *R*_f_: 0.42, 0.35 (dichloromethane–methanol 95 : 5, v/v).

#### 2′-Fluoro-c-di-GMP 9

Fully protected 2′-fluoro-c-di-GMP 8 (50 mg, 0.049 mmol) was co-evaporated with dry toluene (2 × 2 ml) and then dissolved in dry acetonitrile (3.0 ml), followed by addition of 2-nitrobenzaldoxime 13 (0.12 g, 0.72 mmol) and *N*,*N*,*N*′,*N*′-tetramethylguanidine (86 μl, 0.69 mmol). After 16 h the products were evaporated to dryness under reduced pressure and the residue was taken up in concentrated aqueous ammonium hydroxide (33%, 2.0 ml). The reaction mixture was sealed and heated at 55 °C for 15 h. After the mixture was cooled, the products were concentrated to dryness under reduced pressure and then co-evaporated with ethanol (2 × 2 ml). The residue was dissolved in methanol (1.0 ml) and precipitated from diethyl ether (30.0 ml). The product was obtained by centrifugation at 5000 rpm. This precipitation–centrifugation process was repeated 3 more times. The residue was dissolved in HPLC grade water (0.2 ml) followed by addition of 1-butanol (10 × volume). The mixture was vortexed and then frozen. After centrifugation at 10 000 rpm for 10 min, the supernatant was discarded. This 1-butanol extraction step was repeated two more times and the final residue was dissolved in HPLC water (1.0 ml) and passed through an Amberlite (Na^+^ form) cation exchange column (1 × 20 cm). The fractions containing nucleic acid were freeze-dried to give the fully unprotected disodium salt of 2′-fluoro-c-di-GMP 9 as a colourless sponge solid (16 mg, 44%). ESI-MS found [M − H]^−^ = 693.1, C_20_H_21_F_2_N_10_O_12_P_2_^−^ requires 693.07. *δ*_H_[D_2_O, 600 MHz]: 7.84 and 7.83 (2H, H-8), 6.09 (1H, d, *J* = 15.0, H-1′), 6.07 (1H, d, *J* = 15.0, H-1′), 5.48 (2H, two broad signals with *J*_F–H_ = 52.2, H-2′), 5.03 (2H, br, H-3′), 4.38 (2H, d, br, *J* = 7.9, H-4′), 4.31 (2H, d, br, *J* = 12.1, H-5′), 4.02 (2H, dd, *J* = 12.1 and 4.3, H-5′′). *δ*_P_[D_2_O, 242.9 MHz]: −1.40.

#### Antigens and adjuvants

c-di-GMP was synthesized according to procedures reported previously.^10^ Cholera toxin (CT) was purchased from Sigma-Aldrich Canada Ltd (Oakville, Ontario, Canada). The recombinant pneumococcal surface adhesion A (PsaA) of *S. pneumoniae* and *H. pylori* cell-free sonicate extract (HPCE) were prepared as described in details previously.^4,18^ For the preparation of flagellins from *L. monocytogenes* and *C. difficile*, bacterial strains were grown overnight at 37 °C on agar plates. Cells were scraped from surface and resuspended in 10 mM Tris pH 7.4. Flagella were sheared from surface of bacterial cells using a warring blender (4 × 1 min). Bacterial cells were removed by low speed centrifugation and then flagellar filaments were collected from supernatant by ultracentrifugation (100 000 × *g*, 1 hour). Flagellar samples were analysed by SDS-PAGE.

#### Mice

Six to eight-week-old female BALB/c or C57BL/6 mice were purchased from Charles Rivers Laboratories (St. Constant, Quebec). The animals were housed under specific-pathogen-free conditions in a federally licensed small animal containment level 2 facility, and given free access to sterile water and certified mouse chow. The animals were maintained and used in accordance with the recommendations of the Canadian Council on Animal Care Guide to the Care and Use of Experimental Animals. All experimental procedures were approved by the institutional animal care committee (Institute for Biological Sciences, National Research Council Canada, Ottawa, Ontario).

### Intranasal or oral immunization in mice

For intranasal immunization, mice were lightly anesthetized under isofluorane. On day 0, 14 and 21, groups of mice (*n* = 5) were inoculated with 50 μl of various vaccine preparations or controls as detailed in Results section. For oral immunization, various vaccine preparations or controls in 0.5 ml volume were administered by gavage *via* an 18-gauge feeding needle.

At day 28, blood and mucosal samples (fecal pellets or/and vaginal wash) were collected as described elsewhere.^19^ In some experiments, nasal wash samples were also collected at the end of experiment. For both the vaginal and nasal wash, the volume of the lavage fluid recovered from each mouse was recorded. Since the recovery of lavage volumes among different mice in our study were very similar (about 90%), the small variation among individual samples was not adjusted. All samples were stored at −20 °C until assay.

### Enzyme-linked immunosorbent assay (ELISA) for measurement of antigen-specific antibodies in the serum and mucosal samples

Levels of antigen-specific antibodies in serum and mucosal samples were measured by an enzyme-linked immunosorbent assay (ELISA) modified from a previously described procedure.^16^ Briefly, 96-well microplates (Thermo Electron Corporation, Milford, MA) were coated with purified PsaA (5 μg ml^−1^, 100 μl per well), purified flagellins (5 μg ml^−1^, 50 μl per well), or HPCE (20 μg ml^−1^; 50 μl per well) in 0.015 M sodium carbonate and 0.035 M sodium bicarbonate buffer (pH 9.6) at 4 °C overnight. All the subsequent incubations were carried out at room temperature. The wells were blocked by incubation with 5% bovine serum in PBS for 1 h, and then rinsed three times with PBS-0.05% Tween 20. Duplicates of 100 μl pre-diluted samples were added to the wells. Sample dilutions were as follows: 1 : 2 for fecal extracts and nasal washes, 1 : 2000 for serum IgG1, 1 : 500 for serum IgG2a and 1 : 50 for serum IgA. After the plates were incubated for 3 h, alkaline phosphatase-conjugated goat antibodies specific for mouse IgA, IgG1 and IgG2a (all from Caltag Laboratories, Burlingame, CA) were added and incubated for 1 h. Colour reactions were developed by the addition of *p*-nitrophenyl phosphate (*p*NPP) substrate (KPL, Inc., Gaithersburg, MD), and optical density was measured at 405 nm with an automated ELISA plate reader (Multiskan Ascent, Thermo Labsystems, Vantaa, Finland). Pooled samples collected from mice that had been intranasally immunized with the relevant vaccines or from the naïve mice were used as positive or negative controls respectively.

### Intranasal challenge of mice with *S. pneumoniae*

Fresh inocula were prepared for each experiment from a frozen stock of *S. pneumoniae* (type 14). Stock vials of *S. pneumoniae* were thawed and the culture revived on chocolate agar, which was then used to inoculate Todd-Hewitt and Columbia broth supplemented with 1% glucose and 0.1% sodium bicarbonate. The broth culture was incubated in a candle jar at 37 °C for approximately 6 h. The broth culture in mid-logarithmic growth phase was centrifuged at 12 000 × *g* for 15 min, cells were resuspended in PBS and used immediately. Fourteen days after the last immunization, mice were anesthetized and intranasally inoculated with approximately 10^7^ colony-forming units (CFU) *S. pneumoniae* in 50 μl saline. The actual inoculum concentrations were determined by plating 10-fold serial dilutions on chocolate agar. Inoculated mice were sacrificed 3 days later and the nasal cavity was lavaged with 0.5 ml lavage fluid and aliquots (100 μl) of 10-fold serial dilutions of the lavage fluids were cultured, in duplicates, on chocolate agar plates to quantify the number of viable organisms.

### *H. pylori* and oral *H. pylori* infection

The mouse-adapted *H. pylori* SS1 that was established by Lee *et al.*^20^ was used as the challenge strain. Bacteria were grown on brain–heart infusion (BHI) broth supplemented with 5% horse serum under a microaerophilic atmosphere created by a CampyGen generator (Oxoid Ltd, Hampshire, England) at 37 °C for 48 h. The bacterial cells were harvested by centrifugation at 12 000 × *g* for 10 min at 4 °C, washed with PBS three times and then suspended in PBS.

The mice were inoculated orally with 1 × 10^8^ CFU of freshly harvested *H. pylori* in 500 μl BHI broth by using a 18-gauge feeding needle as described previously.^16^ The inoculations were repeated twice (a total of three inoculations) over a period of 5 days. Mice were killed by an overdose of carbon dioxide 4 weeks after the last inoculation, and the serum and stomachs of the animals were collected for analysis. In some experiments, spleens were removed for splenocyte culture and determination of cytokine responses.

### Assessment of *H. pylori* infection

The presence of *H. pylori* infection in individual mice was determined by quantitative bacterial culture as described previously.^16^ Briefly, the mouse stomach was opened longitudinally along the greater curvature and gently washed three times in PBS to remove the stomach contents. The stomach tissue was then homogenized in 5 ml saline with Teflon pestles and glass tubes. The homogenates were serially diluted in saline, and 100 μl of the dilutions were plated onto GSSA (Glaxo Selective Supplement Antibiotics)-supplemented agar plates. The plates were incubated for 96 h at 37 °C under microaerophilic conditions, and the number of colonies were counted and expressed as CFU per stomach. With this method, log_10_ 2.7 CFU per stomach represented the limit of detection.

### Splenocyte culture and cytokine assay

Spleens were aseptically removed and placed in 5 ml Dulbecco's modified Eagle's medium (DMEM). Single cell suspensions were prepared by pressing the spleen through a 100 μm Nylon cell strainer into a sterile Petri dish, using the rubber end of a 1 ml syringe plunger. Erythrocytes from spleens were lysed using an ACK lysis buffer. Cells were washed one time with DMEM and resuspended at a concentration of 3 × 10^7^ cells per ml in DMEM supplemented with 10% fetal bovine serum (FBS). Cells were seeded in duplicate on a 96-well cell culture plate and stimulated with the mixed *H. pylori* antigens (10 μg ml^−1^ each) for 48 h at 37 °C and 5% CO_2_. Culture supernatants were collected and assayed for the concentrations of IFN-γ, IL-2 and IL-17 using Luminex kits.^21^

### Statistical analysis

Data are presented as mean ± standard deviation (SD) for parametric data, unless otherwise indicated. Differences among experimental groups were analyzed by Student's *t*-test or one-way ANOVA followed by Bonferroni or Dunnett's multiple pairwise comparison tests, when appropriated. Differences were considered significant at *P* < 0.05. All statistical analyses were conducted using GraphPad Prism Version 5.0 (GraphPad Software, San Diego, CA).

## Conclusions

The modified H-phosphonate chemistry was found to be suitable for the synthesis of 2′-F-c-di-GMP. This fluorinated c-di-GMP analogue was shown to possess excellent adjuvanticity both intranasally and orally. In this respect, i.n. immunization of BALB/c mice with PsaA adjuvanted with 2′-F-c-di-GMP led to the induction of antigen-specific antibodies both systemically and in the mucosa, and provided protective immunity against *S. pneumoniae* infections. Similar results were seen in C56BL/6 mice orally immunized with *H. pylori* HPCE adjuvanted with 2′-F-c-di-GMP. Furthermore, immunization of C56BL/6 mice with flagillin proteins from *C. difficile* and *L. monocytogenes* adjuvanted with 2′-F-c-di-GMP led to successful induction of antigen-specific antibodies both systemically and mucosally. Mechanistically, this work showed that the protection against *H. pylori* infection induced by oral immunization with 2′-F-c-di-GMP as adjuvant is associated with the enhanced production of antigen-specific IFN-γ. This observation appears to be in contrast to the cytokine induction when CT was used as an adjuvant. In the latter case, production of IL-17 appeared to be more profound, suggesting different mode of activation of the host immune system by 2′-F-c-di-GMP and CT as adjuvants.

## Conflicts of interest

Work described in this manuscript is partially disclosed in WO patent 2015/074145A1 and US patent 10092644.

## Supplementary Material

RA-009-C9RA08310C-s001

## References

[cit1] Chen W., KuoLee R., Yan H. (2010). Vaccine.

[cit2] Libanova R., Becker P. D., Guzman C. A. (2012). Microb. Biotechnol..

[cit3] Zhao L., KuoLee R., Harris G., Tram K., Yan H., Chen W. (2011). Int. Immunopharmacol..

[cit4] Yan H., KuoLee R., Tram K., Qiu H., Zhang J., Patel G. B., Chen W. (2009). Biochem. Biophys. Res. Commun..

[cit5] Guo F., Li Q., Zhou C. (2017). Org. Biomol. Chem..

[cit6] Müller K., Faeh C., Diederich F. (2007). Science.

[cit7] Gillis E. P., Eastman K. J., Hill M. D., Donnelly D. J., Meanwell N. A. (2015). J. Med. Chem..

[cit8] Richardson P. (2016). Expert Opin. Drug Discovery.

[cit9] Yerien D. E., Bonesi S., Postigo A. (2016). Org. Biomol. Chem..

[cit10] Yan H., Anguilar A. L. (2007). Nucleosides, Nucleotides Nucleic Acids.

[cit11] Yan H., Wang X., KuoLee R., Chen W. (2008). Bioorg. Med. Chem. Lett..

[cit12] Gray P. M., Forrest G., Wisniewski T., Porter G., Freed D. C., DeMartino J. A., Zaller D. M., Guo Z., Leone J., Fu T. M., Vora K. A. (2012). Cell. Immunol..

[cit13] Ebensen T., Schulze K., Riese P., Link C., Morr M., Guzman C. A. (2007). Vaccine.

[cit14] Hu D. L., Narita K., Hyodo M., Hayakawa Y., Nakane A., Karaolis D. K. R. (2009). Vaccine.

[cit15] Miquel-Clopés A., Bentley E. G., Stewart J. P., Carding S. R. (2019). Clin. Exp. Immunol..

[cit16] Chen W., Shu D., Chadwick V. S. (2001). J. Gastroenterol. Hepatol..

[cit17] Larussa T., Leone I., Suraci E., Imeneo M., Luzza F. (2015). J. Immunol. Res..

[cit18] Chen W., Shu D., Chadwick V. S. (2000). J. Gastroenterol. Hepatol..

[cit19] Patel G. B., Zhou H., Ponce A., Chen W. (2007). Vaccine.

[cit20] Lee A., O'Rourke J., De Ungria M. C., Robertson B., Daskalopoulos G., Dixon M. F. (1997). Gastroenterologia.

[cit21] Harris G., KuoLee R., Xu H. H., Chen H. (2019). Sci. Rep..

