# Supplementary material for: 2′-Fluoro-c-di-GMP as an oral vaccine adjuvant
Source: RSC Adv. 2019 Dec 16;9(71):41481–9. doi: 10.1039/c9ra08310c (PMC9076492; doi:10.1039/c9ra08310c)
Supplement: RA-009-C9RA08310C-s001 [file RA-009-C9RA08310C-s001.pdf]

Electronic Supplementary Material (ESI) for RSC Advances.
This journal is © The Royal Society of Chemistry 2019

# 2’-Fluoro-c-di-GMP as an oral vaccine adjuvant

Jia Li,[a] Rhonda Kuo Lee,[b] Wangxue Chen,[b,c]* Hongbin Yan[a]*

[a]*Department of Chemistry, Brock University, 1812 Sir Isaac Brock Way, St. Catharines, ON L2S 3A1, Canada Email: tyan@brocku.ca*

[b]*Human Health and Therapeutics Research Center, National Research Council of Canada, 100 Sussex Dr. Ottawa, ON K1A 0R6, Canada. Email: wangxue.chen@nrc-cnrc.gc.ca*

[c]*Department of Biological Sciences, Brock University, 1812 Sir Isaac Brock Way, St. Catharines, ON L2S 3A1, Canada.*

(a)

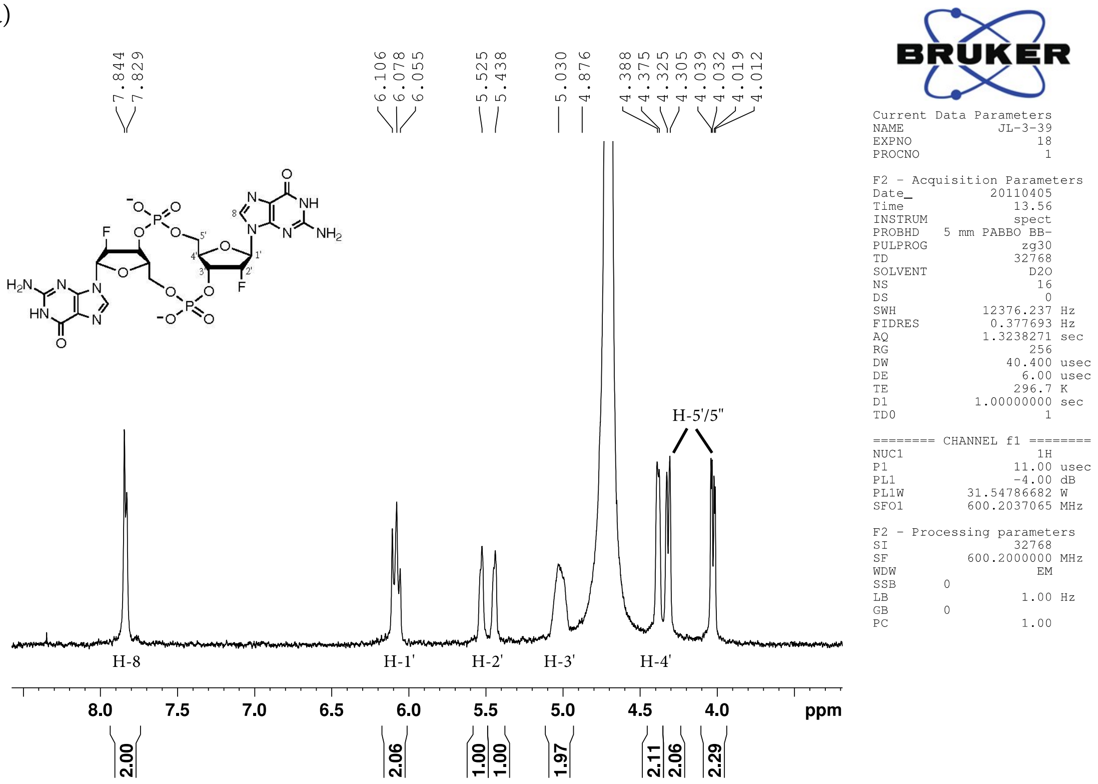
7.844
7.829
6.106
6.078
6.055
5.525
5.438
5.030
4.876
4.388
4.375
4.325
4.305
4.039
4.032
4.019
4.012
H-8
H-1'
H-2'
H-3'
H-4'
H-5'/5"
8.0 7.5 7.0 6.5 6.0 5.5 5.0 4.5 4.0 ppm
2.00
2.06
1.00
1.00
1.97
2.11
2.06
2.29
BRUKER
Current Data Parameters
NAME JL-3-39
EXPNO 18
PROCNO 1
F2 - Acquisition Parameters
Date_ 20110405
Time 13.56
INSTRUM spect
PROBHD 5 mm PABBO BB-
PULPROG zg30
TD 32768
SOLVENT D2O
NS 16
DS 0
SWH 12376.237 Hz
FIDRES 0.377693 Hz
AQ 1.3238271 sec
RG 256
DW 40.400 usec
DE 6.00 usec
TE 296.7 K
D1 1.00000000 sec
TD0 1
======== CHANNEL f1 ========
NUC1 1H
P1 11.00 usec
PL1 -4.00 dB
PL1W 31.54786682 W
SFO1 600.2037065 MHz
F2 - Processing parameters
SI 32768
SF 600.2000000 MHz
WDW EM
SSB 0
LB 1.00 Hz
GB 0
PC 1.00

(b)

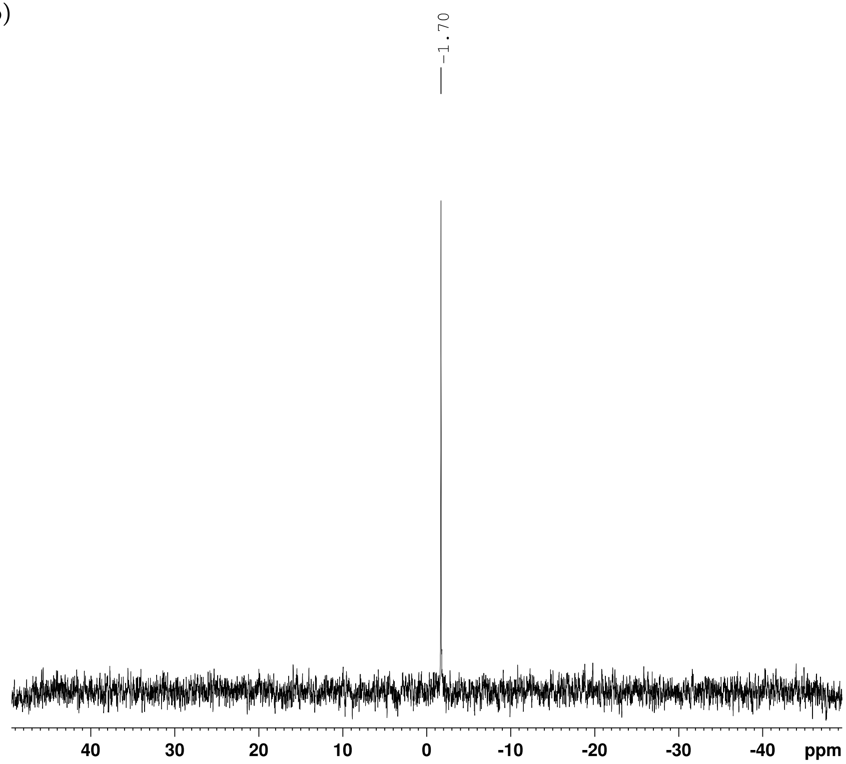


```
Current Data Parameters
NAME              JL-3-39
EXPNO                  19
PROCNO                  1

F2 - Acquisition Parameters
Date_          20110405
Time              14.00
INSTRUM           spect
PROBHD   5 mm PABBO BB-
PULPROG         zgig30
TD                32768
SOLVENT             D2O
NS                   64
DS                    0
SWH           24038.461 Hz
FIDRES         0.733596 Hz
AQ            0.6815744 sec
RG              23170.5
DW               20.800 usec
DE                 6.00 usec
TE                296.8 K
D1           2.00000000 sec
D11          0.03000000 sec
TD0                   1

======== CHANNEL f1 ========
NUC1                31P
P1                11.00 usec
PL1               -1.00 dB
PL1W        80.00562286 W
SFO1        242.9654130 MHz

======== CHANNEL f2 ========
CPDPRG[2        waltz16
NUC2                 1H
PCPD2             70.00 usec
PL2               -4.00 dB
PL12              12.07 dB
PL2W        31.54786682 W
PL12W        0.77977633 W
SFO2        600.2024008 MHz

F2 - Processing parameters
SI                16384
SF          242.9654130 MHz
WDW                  EM
SSB      0
LB                 5.00 Hz
GB       0
PC                 1.40
```

(c)

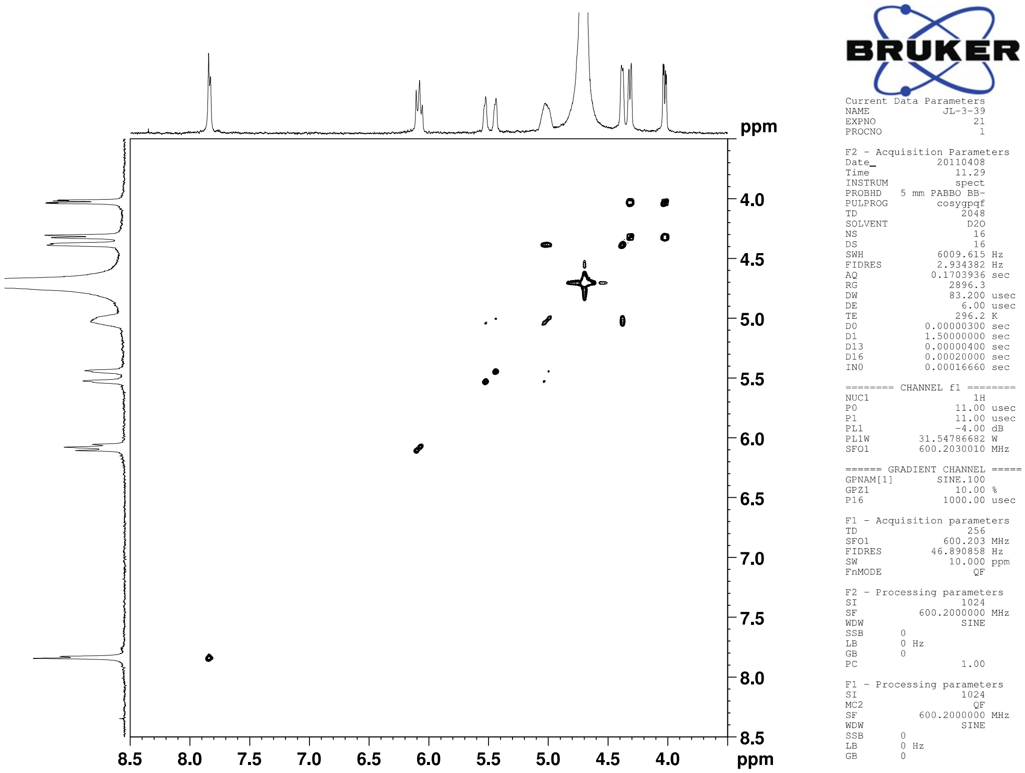


**Figure S1.** NMR spectra of the fully-deprotected 2'-F-c-di-GMP in $D_2O$. a) $^{1}H$ NMR, b) $^{31}P$ and c) COSY.

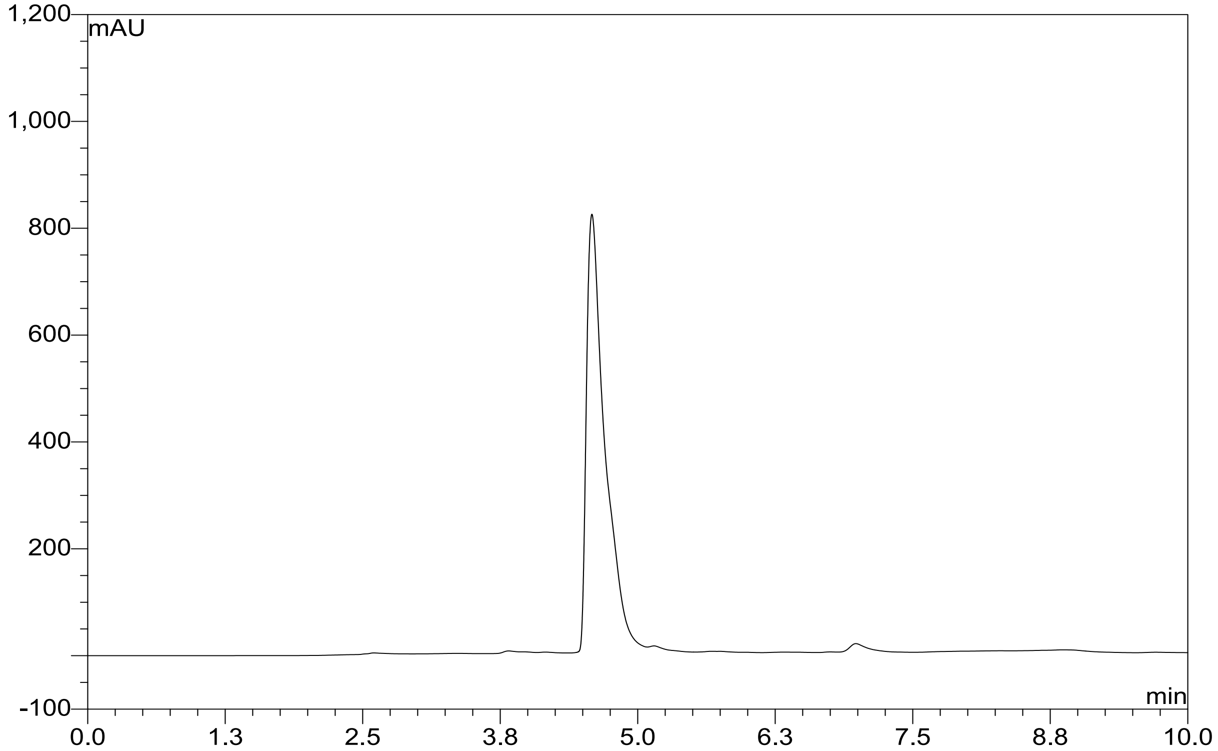


**Figure S2.** HPLC profile of the fully deblocked 2’-F-c-di-GMP. The column (Clarity 3 μm oligo reverse phase, 4.6×50 mm) was eluted with a linear gradient of acetonitrile – triethylammonium acetate (20 mM, pH 7.00) (0:100 to 15:85, v.v, over 10 min  at 0.7 ml/min).

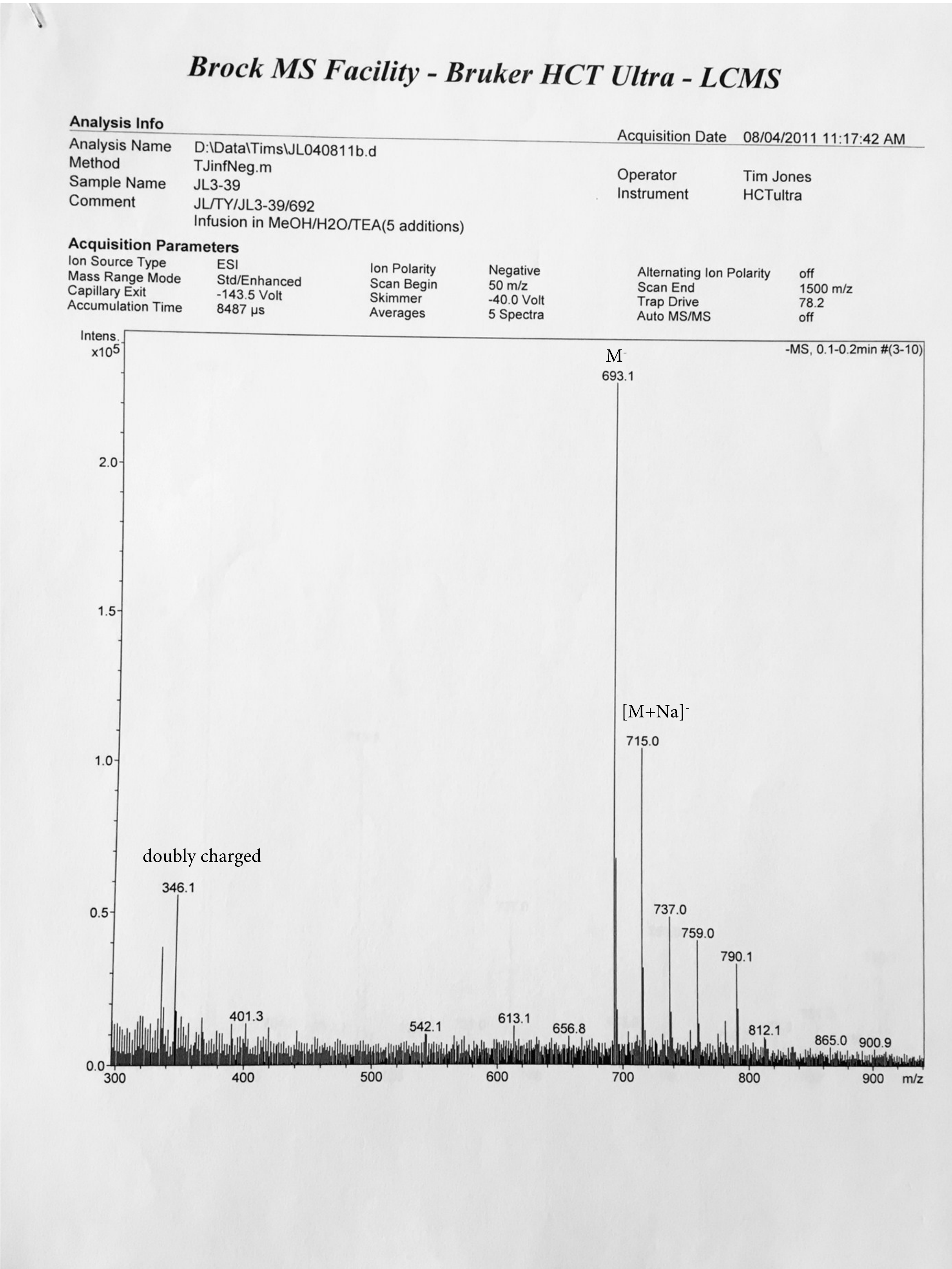


**Figure S3**. Mass spectrum of 2'-F-cdi-GMP under ESI negative mode.
